# Comprehensive analysis of GSEC/miR-101-3p/SNX16/PAPOLG axis in hepatocellular carcinoma

**DOI:** 10.1371/journal.pone.0267117

**Published:** 2022-04-28

**Authors:** Shangshang Hu, Jinyan Zhang, Guoqing Guo, Li Zhang, Jing Dai, Yu Gao

**Affiliations:** 1 Research Center of Clinical Laboratory Science, School of Laboratory Medicine, Bengbu Medical College, Bengbu, China; 2 School of Life Science, Bengbu Medical College, Bengbu, China; 3 Anhui Province Key Laboratory of Translational Cancer Research, Bengbu Medical College, Bengbu, China; Indian Institute of Technology Delhi, INDIA

## Abstract

Hepatocellular carcinoma (HCC) is one of the most lethal malignancies. A growing number of studies have shown that competitive endogenous RNA (ceRNA) regulatory networks might play important roles during HCC process. The present study aimed to identify a regulatory axis of the ceRNA network associated with the development of HCC. The roles of SNX16 and PAPOLG in HCC were comprehensively analyzed using bioinformatics tools. Subsequently, the “mRNA-miRNA-lncRNA” model was then used to predict the upstream miRNAs and lncRNAs of SNX16 and PAPOLG using the miRNet database, and the miRNAs with low expression and good prognosis in HCC and the lncRNAs with high expression and poor prognosis in HCC were screened by differential expression and survival analysis. Finally, the risk-prognosis models of ceRNA network axes were constructed by univariate and multifactorial Cox proportional risk analysis, and the immune correlations of ceRNA network axes were analyzed using the TIMER and GEPIA database. In this study, the relevant ceRNA network axis GSEC/miR-101-3p/SNX16/PAPOLG with HCC prognosis was constructed, in which GSEC, SNX16, and PAPOLG were highly expressed in HCC with poor prognosis, while miR-101-3p was lowly expressed in HCC with good prognosis. The risk-prognosis model predicted AUC of 0.691, 0.623, and 0.626 for patient survival at 1, 3, and 5 years, respectively. Immuno-infiltration analysis suggested that the GSEC/miR-101-3p/SNX16/PAPOLG axis might affect macrophage polarization. The GSEC/miR-101-3p/SNX16/PAPOLG axis of the ceRNA network axis might be an important factor associated with HCC prognosis and immune infiltration.

## Introduction

Primary liver cancer is one of the most common malignancies, and about 80% of primary liver cancer is hepatocellular carcinoma (HCC) [1]. After decades of development, the treatment modalities for HCC have been greatly improved, including in early diagnosis, surgery, chemotherapy, liver transplantation, and drug therapy [2]. However, the prognosis of HCC patients is still inferior, and it is estimated that the survival rate of HCC patients at two years is less than 50%, while the survival rate at five years is about 10% [3]. In addition, although numerous studies have extensively investigated the relationship between abnormal gene expression and the development of HCC, the specific mechanism of HCC is uncertain. Therefore, it is essential to identify the mechanism of occurrence related to the prognosis of HCC.

Long-stranded non-coding RNA (lncRNA) is a class of RNA that exceeds 200 nt [4]. Various researches indicated that abnormal expression of lncRNAs is highly correlated to the prognosis and metastasis of HCC [5, 6]. MicroRNA (miRNA) is a class of endogenous, single-stranded, non-coding RNAs of 19–25 nt length [7], in which its prominent role is to bind to the 3’ untranslated region of its target mRNA, thereby inhibiting gene expression [8]. The interactions of mRNA, miRNA, and lncRNA play multiple roles in the development of HCC. LncRNA could act as an endogenous molecular sponge competing for binding miRNA and regulating the expression level of mRNA. The ceRNA (competing endogenous RNA) actually refers to the regulatory mechanism between different kinds of RNAs, including lncRNA, miRNA and mRNA [9]. The ceRNA network also plays a substantial regulatory role in the progression of HCC, wherein cytoplasmic species lncRNA acts as an endogenous molecular sponge competing for binding miRNA and regulating the expression level of mRNA [10]. For example, Zhikui Liu *et al*. reported that the lncRNA RNA AGAP2-AS1 acted as ceRNA to upregulate ANXA11 expression by miR-16-5p and promoted the progression of HCC [11].

Sorting nexins (SNXs) are a family of phosphatidylinositol-binding proteins defined by a common PX domain, which plays a key role in membrane transport [12]. Sorting nexin 16 (SNX16), a member of the Sorting nexins (SNXs) family, is associated with hepatitis C virus replication [13] and the transport of E-calmodulin [14]. In a recent study, SNX16 was highly expressed in colorectal cancer and activated the c-Myc signaling pathway by upregulating eEF1A2, thereby promoting colorectal cancer development [15]. Poly(A) polymerase gamma (PAPOLG), a member of the poly(A) polymerase (PAP) family, plays an important role in mRNA stability and translational modifications [16]. A recent report showed that PAPOLG cloud be overexpressed as a proto-oncogene in transformed follicular lymphoma (tFL) and follicular lymphoma (FL), and played an important role in FL to tFL transformation [17]. However, there was no study on the effects SNX16 or PAPOLG in the progression of HCC.

**Fig 1:**
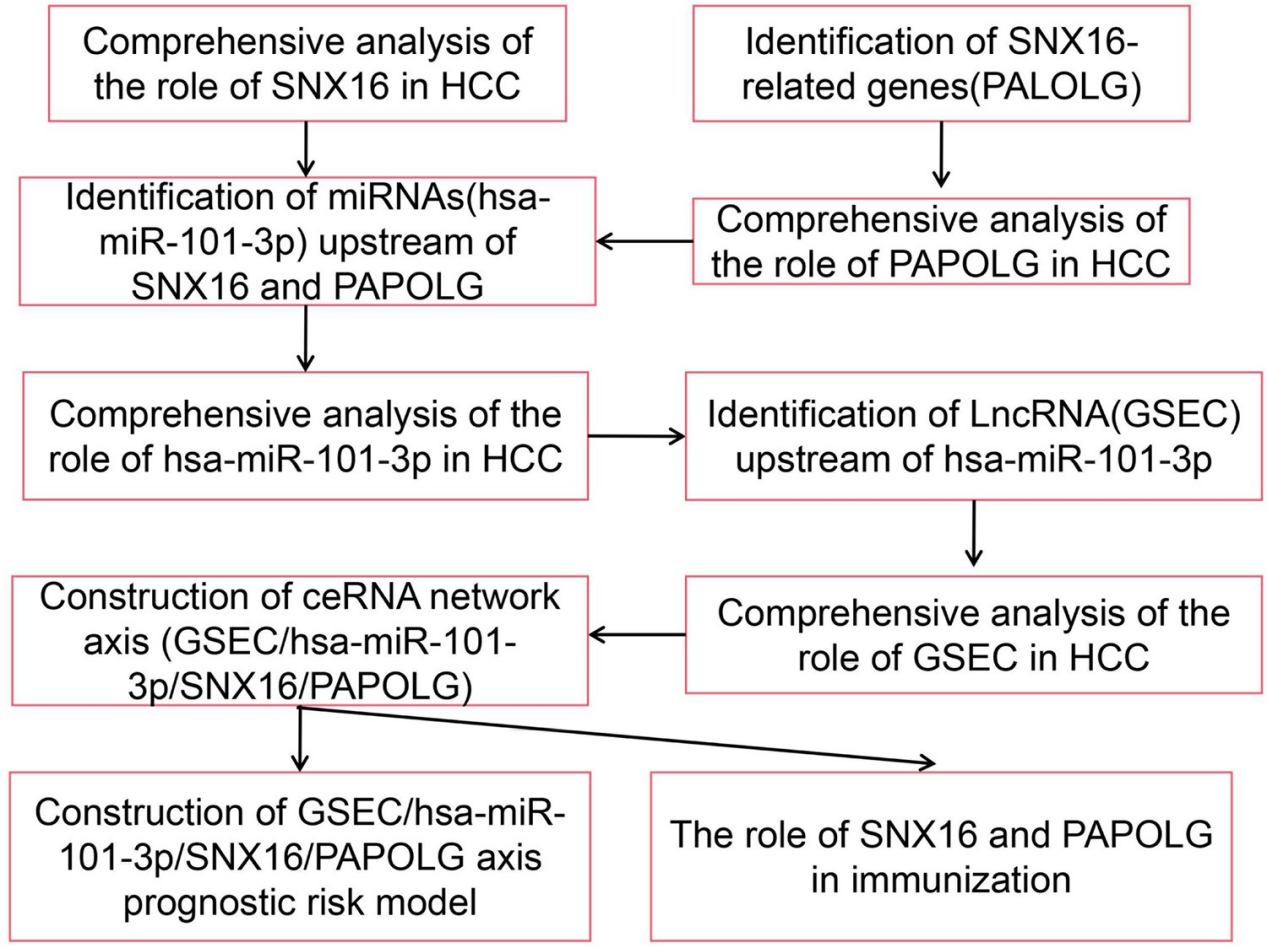
Flow chart of the analysis of this study.

In the current study, we investigated the effect of competitive endogenous RNA (ceRNA) regulatory networks in HCC. The flowchart of this study was described Fig 1. Firstly, the expression profiles of SNX16 (Sorting Nexin 16) and PAPOLG (Poly(A) Polymerase Gamma) in HCC were analyzed comprehensively. And then, the ceRNA network axis associated with SNX16 and PAPOLG was constructed. Finally, the prognostic model of the ceRNA network axis was constructed by univariate and multifactorial Cox proportional risk analysis, and the immune relationship of the ceRNA network axis was analyzed using the TIMER database and GEPIA database. Here, the ceRNA network was also verified by using different liver cell lines.

## Materials and methods

### Data acquisition

Expression profile data and clinical data for HCC were acquired from the TCGA database (The Cancer Genome Atlas). Expression profile data included mRNA (tumor: n = 374, normal: n = 50), miRNA (tumor: n = 375, normal: n = 50) and lncRNA (tumor: n = 374, normal: n = 50). And expression data also included a total of 50 pairs of HCC cancer tissues and matched adjacent normal tissues. Clinical data were retained for samples containing survival time, survival status, age, gender, grade, TNM stage, invasion depth (T), lymph node metastasis (N), and distant metastasis (M) (mRNA/lncRNA: n = 235, miRNA: n = 237). The diagnostic criteria of HCC patients and case sources were referred to TCGA database. As previously reported, each HCC patient had an image of a full scanned H&E slide available for review, in addition to each case being independently reviewed by at least 3 liver pathologists [18]. The clinicopathological characteristics statistics of HCC patients were listed in S1 and S2 Tables. The expression data of GSE25097 (platform: GPL10687) (tumor: n = 268, normal: n = 249) [19] were acquired from the Gene Expression Omnibus (GEO) database to further validate the results based on TCGA. At the protein expression level, data from the Human Protein Atlas (HPA) (https://www.proteinatlas.org/) were used to verify the expression of PAPOLG and SNX16. Visualization of variance analysis using the R software ggpubr package.

### Screening for SNX16-related genes

Based on GTEx (The Genotype-Tissue Expression) and TCGA data, the top 40 Gene Expression Profiling Interactive Analysis [20] (GEPIA) website Similar Genes module was obtained genes related to SNX16. The list of top 40 genes related with SNX16 in the UALCAN [21] database was also obtained. Commonly related genes were obtained by the Venn diagram. The Venn diagrams were drawn using the R software VennDiagram package. Finally, the correlation between SNX16 and PAPOLG was visualized by R software ggplot2 package, ggpubr package and ggExtra package.

### Related miRNA and lncRNA prediction and ceRNA network construction

The online tool of miRNet (https://www.mirnet.ca/miRNet/home.xhtml) [22] was used to predict miRNAs related of SNX16 and PAPOLG and to screen out miRNAs, which were significantly differentially expressed and had a good prognosis. And then, miRNet online tool was also used to predict related lncRNAs and to screen out lncRNAs, which were significantly differentially expressed and had a poor prognosis. Finally, ceRNA network construction was performed and visualized with Cytoscape software was used for visualization. lncLocator website was used to identify the positioning of lncRNAs in cells [23]. The Encyclopedia of RNA Interactomes (ENCORI) database was used to identify the sites of action between mRNA miRNA and lncRNA [24].

### Survival analysis and construction of ceRNA network axis risk prognostic model

HCC samples (mRNA/lncRNA: n = 370, miRNA: n = 371) containing survival time and survival status were acquired from the TCGA database. The HCC patients were divided into two groups based on median gene expression values, followed by survival analysis using the R software survminer package and survivor package. The best risk-prognosis model was constructed using the R software survivor package and multifactorial Cox analysis according to the Akaike information criterion (AIC). Risk value = GSEC-coefficient * GSEC-expression + PAPOLG-coefficient * PAPOLG-expression. Based on the median risk value, HCC patients were divided into two groups, and survival analysis of the risk model was performed using the R software survminer package and survival package. The R software timeROC package to plot the receiver operating characteristic curve (ROC) curve and calculate the area under the curve (AUC) to estimate the accuracy of the prognosis of this risk model. Univariate and multifactor COX regression analyses were performed on clinical characteristics based on the risk model.

### Gene set enrichment analysis (GSEA)

Based on the median SNX16 and PAPOLG expression values, HCC patients were analyzed within two groups (high and low expression groups). KEGG enrichment analysis was performed on the high expression group using GSEA 4.1.0 software, with setting conditions: nominal (NOM) P-value < 0.01, false discovery rate (FDR) < 0.05.

### Analysis of SNX16 and PAPOLG expression and immune infiltration

TIMER online tool is a comprehensive database of multiple data analyzing multiple cancers associated with immune infiltration [25]. The TIMER database was used to explore the relationship between SNX16 and PAPOLG expression and immune cell infiltrates, including B cells, CD8+ T cells, CD4+ T cells, macrophages, neutrophils, and dendritic cells. And then, the correlation of SNX16 and PAPOLG expression with M2 and tumor-associated macrophages (TAMs) markers was analyzed in this study.

### Cell culture and quantitative real time RT-PCR (qRT-PCR)

Four HCC cell lines (SMMC- 7721, HUH-7, MHCC97-H, and SNU449 HCC) and one normal hepatocyte cell line (WRL-68) were cultured in DMEM medium with antibodies of penicillin and streptomycin, and 10% fetal bovine serum. After conditioning the cells to the appropriate concentration, the cells were cultured at 37°C and 5% CO2. Total RNA (including small miRNA) was extracted using a TRIzol kit, and the total RNA was directly amplified by TransScript^®^ Green One-Step qRT-PCR SuperMix (Transgen, Beijing, China). GSEC, SNX16, and PAPOLG were amplified using GAPDH as the internal reference gene. And miR-101-3p was amplified using U6 as the internal reference gene. RT-qPCR was performed on the amplified products using the LightCycler® 96 SW 1.1 real-time PCR system (Novozymes Biotechnology Co., Ltd.). Experiments were performed with three replicates per sample and repeated three times. Relative gene expression was measured by the 2^-ΔΔCt^ method. The primer sequences for qRT-PCR were listed in Table 1.

**Table 1:** Primer sequences for qRT-PCR.

RNA name	Primer sequence (5’-3’)
**GAPDH**	Forward primer: ATTGAAAATTCAGGATGGGCTTTT
	Reverse primer: GTTTCTGGGCTTCTCTTTGGACTC
**GSEC**	Forward primer: CACATGGTATTCAGGGTCCGATA
	Reverse primer: ACCCAAAGGTCCAAGTTTTCCTT
**SNX16**	Forward primer: AGGGCCAGTTAGAAGACTCAA
	Reverse primer: TGAGGGGACTGCTACAGACAG
**PAPOLG**	Forward primer: TGTCTCTGGATAGCAGTTGTCTGG
	Reverse primer: TTCGTCCTACTACGGTAGGAATGG
**U6**	Forward primer: CTCGCTTCGGCAGCACA
	Reverse primer: AACGCTTCACGAATTTGCGT
**miR-101-3p**	Forward primer: GGTCACTAAGGCGGT
	Reverse primer: CAGTCGTTGCGTCGGAGT

### Statistical analysis

All the above analyses were based on R software version 4.0.3. Differential expression and paired differences analyses were carried using independent samples t-test and paired t-test, respectively. Spearman correlation coefficient was used to analyze the associations between two gene expressions, the Wilcoxon test was used to analyze the relationship between gene expression and clinical variables, and survival analysis was performed using Kaplan-Meier analysis. Setting p<0.05 was statistically significant.

## Results

### Correlation of SNX16 expression with survival and clinical variables in HCC patients

**Fig 2:**
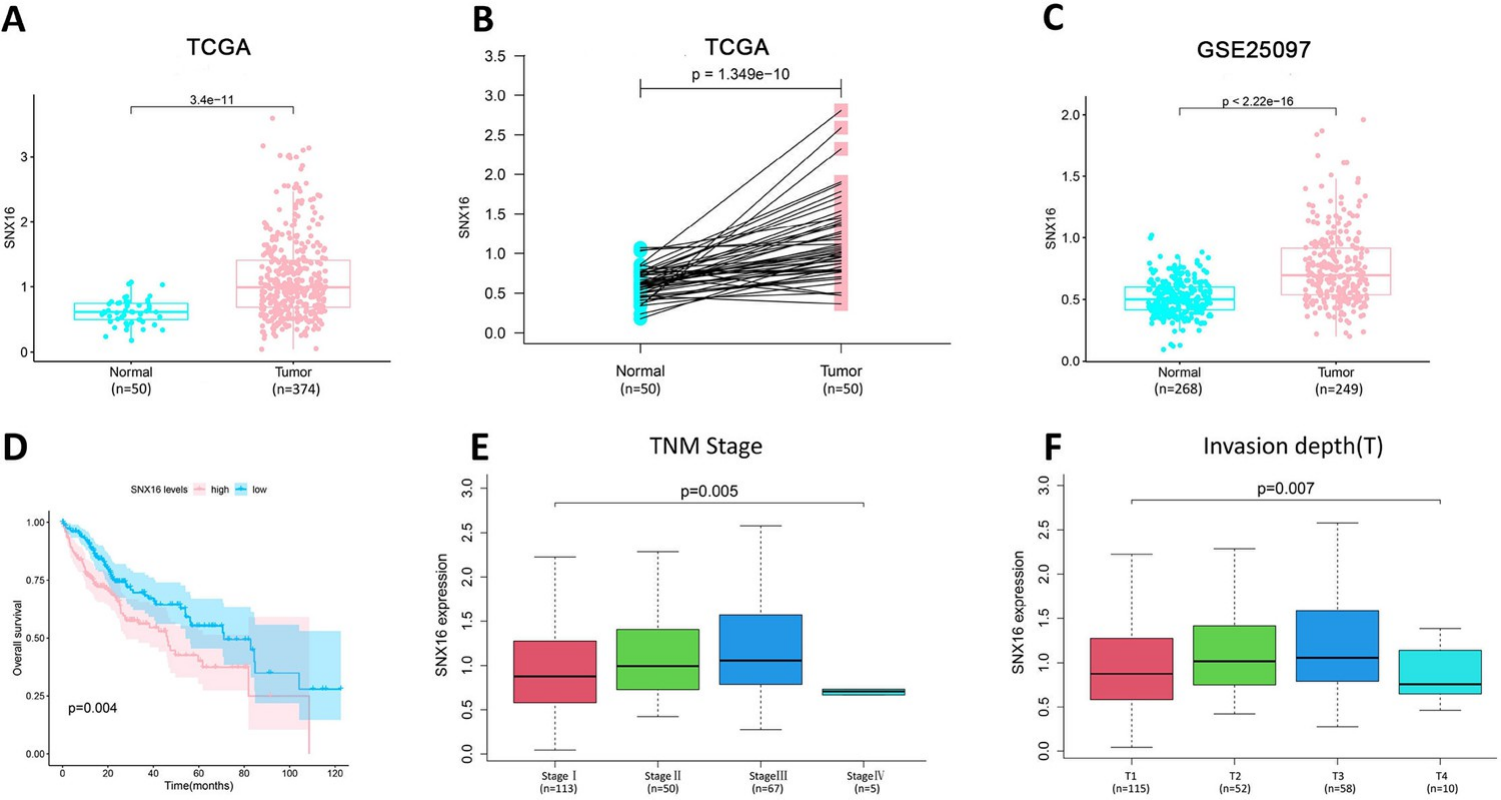
The pro-carcinogenic role of SNX16 in HCC. (A) Differential expression of SNX16 in HCC based on TCGA data. (B) Differential expression of SNX16 in paraneoplastic and HCC tissues based on TCGA data. (C) Differential expression of SNX16 in dataset GSE25097. (D) Analysis of overall survival of SNX16 in HCC patients. (E) Correlation of SNX16 expression with TNM stage. (F) Correlation of SNX16 expression with invasion depth.

Based on TCGA and GEO databases, we analyzed the expression profile of SNX16 in HCC. In the TCGA database, the mRNA expression level of SNX16 was remarkably higher in HCC tissues than normal liver tissues (Fig 2A). SNX16 mRNA expression levels were also remarkably higher in HCC tissues compared to paraneoplastic tissues (Fig 2B). In this study, the expression level of SNX16 mRNA in HCC was further verified using GEO data GSE25097. The data showed that the expression of SNX16 mRNA was also significantly higher in HCC tissues than that in normal liver tissues (Fig 2C). Based on the immunohistochemical picture of HCC from the HPA database, the protein level of SNX16 was dysregulated in HCC samples (S1 Fig), and the immunohistochemical information of HCC patients was listed in S1 Table. According to the Kaplan-Meier survival curve, the overall survival of patients in the high SNX16 expression group in HCC patients was remarkably lower than that of patients in the low SNX16 expression group (Fig 2D). Finally, we combined the SNX16 expression with clinical variables, and found that SNX16 expression was positively correlated with TNM stage (I-III) and Invasion depth (T1-T3) (Fig 2E and 2F). Together, the above results suggest that SNX16 plays an important role in HCC.

### Screening for genes associated with SNX16 and analysis

**Fig 3:**
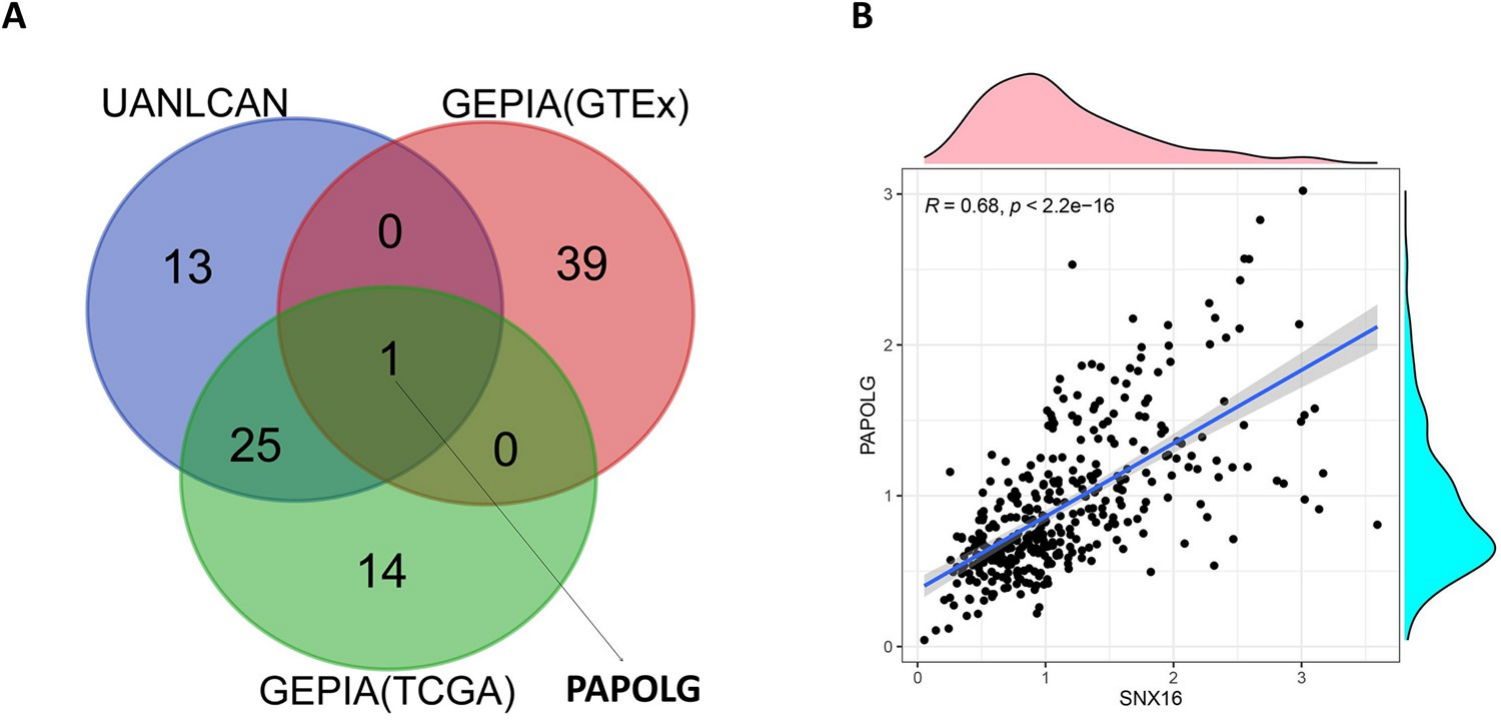
Screening of genes associated with SNX16. (A) Venn diagram showing SNX16-related genes shared by GEPIA(GTEx) database, TCGA database data, and UALCAN. (B) correlation analysis of SNX16 with PAPOLG expression.

**Fig 4:**
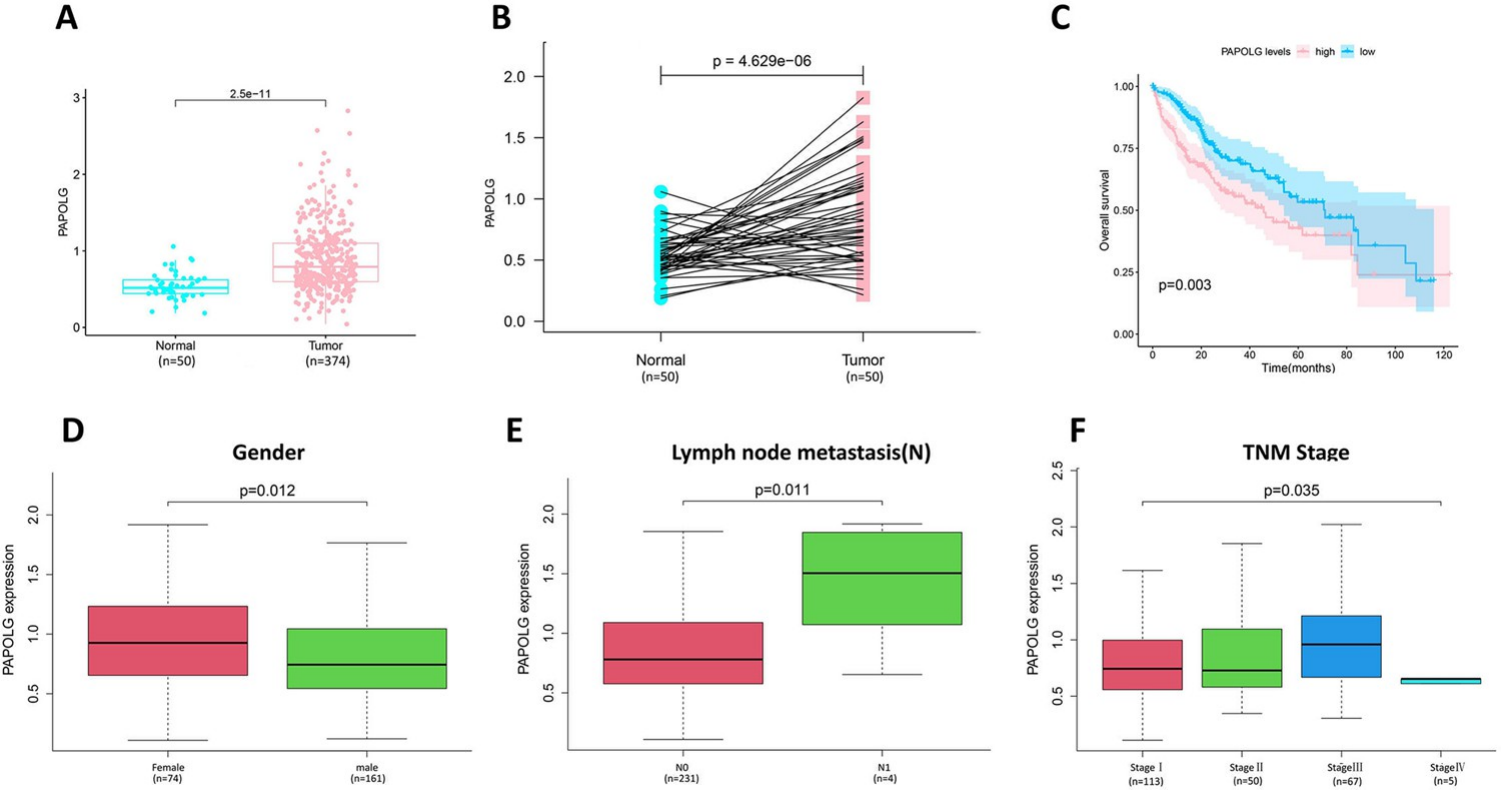
Pro-carcinogenic effect of PAPOLG in HCC. (A) Differential expression of PAPOLG in HCC. (B) Differential expression of PAPOLG in paraneoplastic tissue and HCC tissue. (C) Analysis of overall survival rate of PAPOLG in HCC patients. Correlation of PAPOLG expression with gender (D), lymph node metastasis (E), and TNM stage (F).

Based on the GTEx database and TCGA database data in the GEPIA website, the top forty genes associated with SNX16 in these two databases were obtained, and the top forty genes associated with SNX16 in the UALCAN database were also obtained. The commonly related gene—PAPOLG was obtained (Fig 3A). The correlation between SNX16 and PAPOLG was verified using the TCGA expression data. As shown in Fig 3B, there was a strong relation between SNX16 and PAPOLG (r = 0.68, p<0.05). Subsequently, further analysis of the role of PAPOLG in HCC indicated that PAPOLG was highly expressed in HCC (Fig 4A and 4B), and had a poor prognosis (Fig 4C). Immunohistochemical results at the protein level showed the same dysregulation of PAPOLG expression (S1 Fig) (S3 Table). Combined with clinical variables, PAPOLG expression relation with gender (Fig 4D), Lymph node metastasis (Fig 4E), and TNM stage (Fig 4F). Next, the ceRNA network of SNX16 associated with PAPOLG was constructed.

### Prediction and screening of miRNAs related of SNX16 and PAPOLG

**Fig 5:**
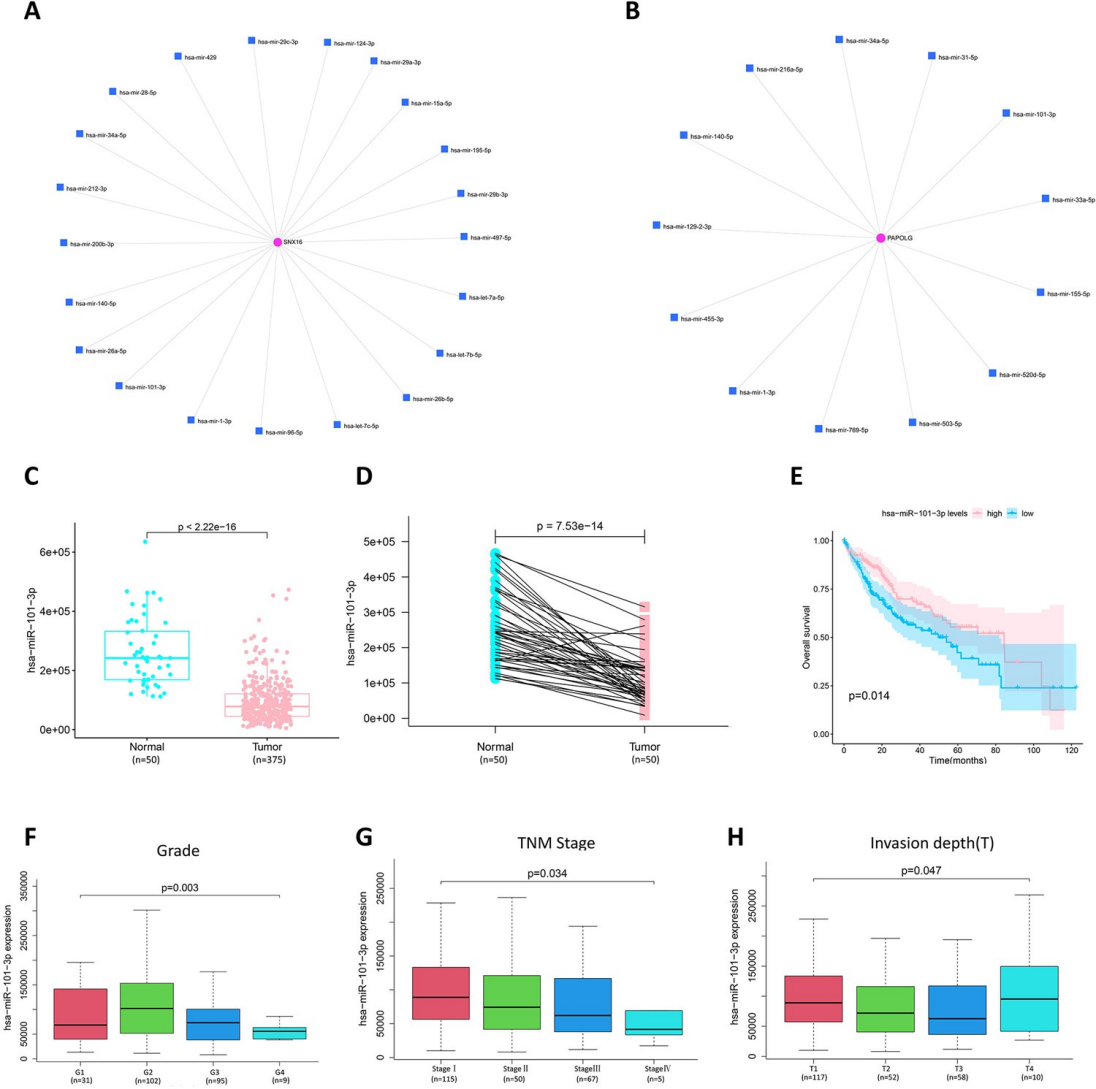
Screening of SNX16 and PAPOLG related miRNA (miR-101-3p) and miR-101-3p for oncogenic effects in HCC. (A) the list of SNX16 related miRNA. (B) the list of PAPOLG related miRNA. (C) Differential expression of miR-101-3p. (D) Differential expression of miR-101-3p in paraneoplastic versus HCC tissues. (E) Analysis of overall survival of miR-101-3p expression in HCC patients. miR-101-3p expression and Grade (F), TNM stage (G), and invasion depth (H) of correlation.

The miRNet online tool was used to predict miRNAs related to SNX16 and PAPOLG, and there was a total of 34 related miRNAs (Fig 5A and 5B). Based on the opposite relationship between miRNAs and target gene mRNAs, miRNAs with low expression and good prognosis in HCC were therefore screened. The commonly related miRNA (miR-101-3p) of SNX16 and PAPOLG was finally screened, and the differential expression and survival analysis of other miRNAs were shown in S3 Fig. The expression level of miR-101-3p was significantly lower in HCC tissues (Fig 5C and 5D). Moreover, the lower expression of miR-101-3p was remarkably related to shorter overall survival in HCC patients (Fig 5E). The miR-101-3p expression was combined with clinical variables, and miR-101-3p expression was found to correlate with Grade (Fig 5F), TNM stage (Fig 5G), and invasion depth (Fig 5H), especially miR-101-3p expression related with TNM stage (I-IV) in a negative correlation. We identified miR-101-3p as the central miRNA.

### Prediction and screening of lncRNA related of miR-101-3p

**Fig 6:**
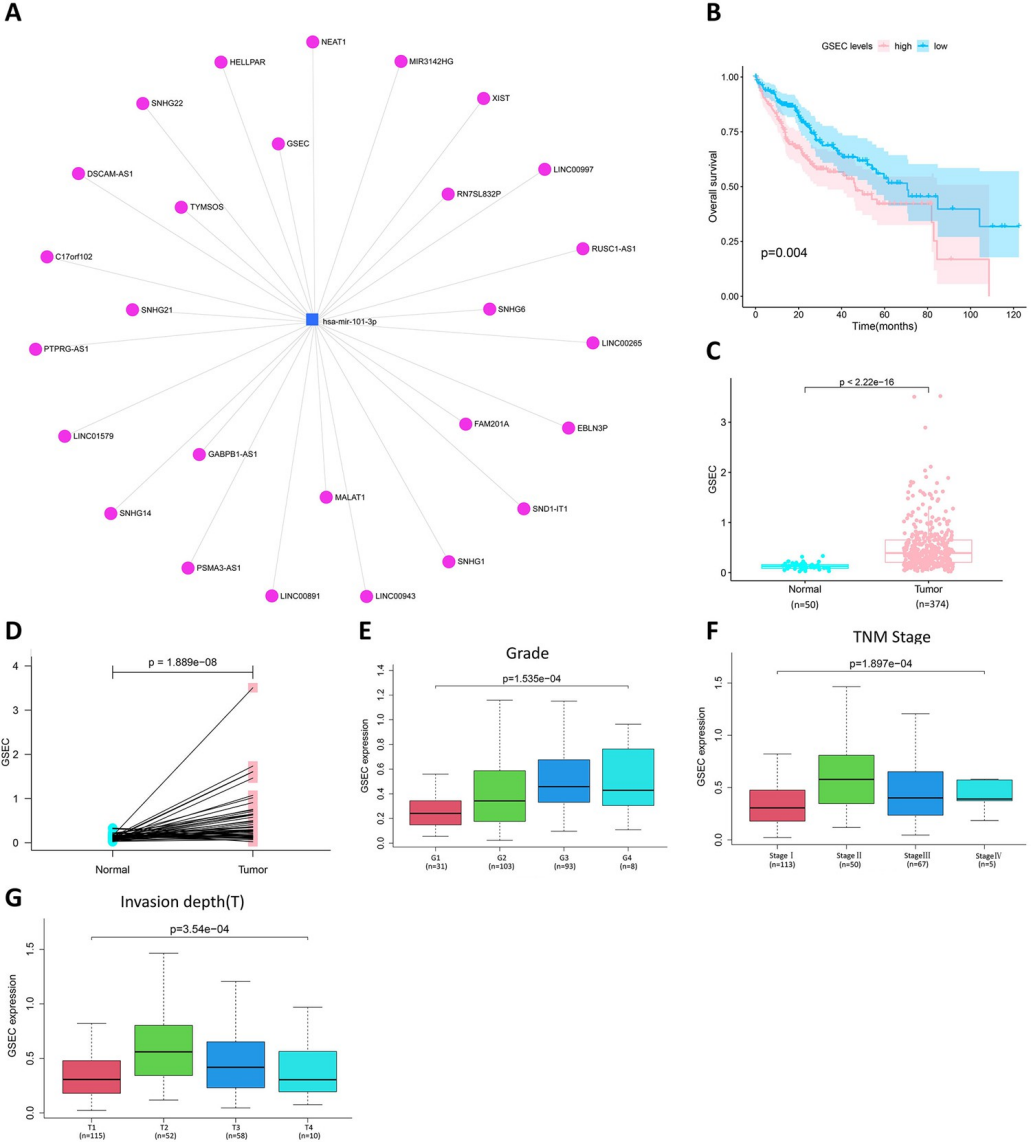
Screening of miR-101-3p related LncRNA (GSEC) and the pro-carcinogenic effect of GSEC in liver cancer. (A) the list of lncRNA related of miR-101-3p. (B) Analysis of overall survival of GSEC in HCC patients. (C) Differential expression of GSEC in HCC. (D) Differential expression of GSEC in paraneoplastic and HCC tissues. Correlation of GSEC expression with grade (E), TNM stage (F), and invasion depth (G).

It is well known that lncRNAs can act as competing endogenous RNAs (ceRNAs) to adjust mRNAs by acting on miRNAs [26]. Therefore, lncRNAs related to miR-101-3p were predicted by the miRNet online website, and a total of 27 lncRNAs were screened (Fig 6A). Based on the ceRNA hypothesis theory, there is a reverse relationship between lncRNAs and miRNAs. Therefore, differential expression and survival analyses were performed on these 27 lncRNAs to screen out lncRNAs with high expression and poor prognosis in HCC. Finally, only lncRNA GSEC (G-quadruplex-forming sequence containing lncRNA) was qualified for the conditions and hypotheses of the ceRNA theory. Moreover, the higher expression of GSEC was remarkably related to shorter overall survival in HCC patients (Fig 6B), and the expression level of GSEC was significantly higher in HCC tissues (Fig 6C and 6D). The differential expression and survival analyses of the other 26 lncRNAs were shown in S4 Fig. GSEC expression was combined with clinical variables, and GSEC expression was found to be associated with Grade (Fig 6E), TNM stage (Fig 6F), and invasion depth (Fig 6G).

### Construction of ceRNA network axis GSEC/miR-101-3p/SNX16/PAPOLG

**Fig 7:**
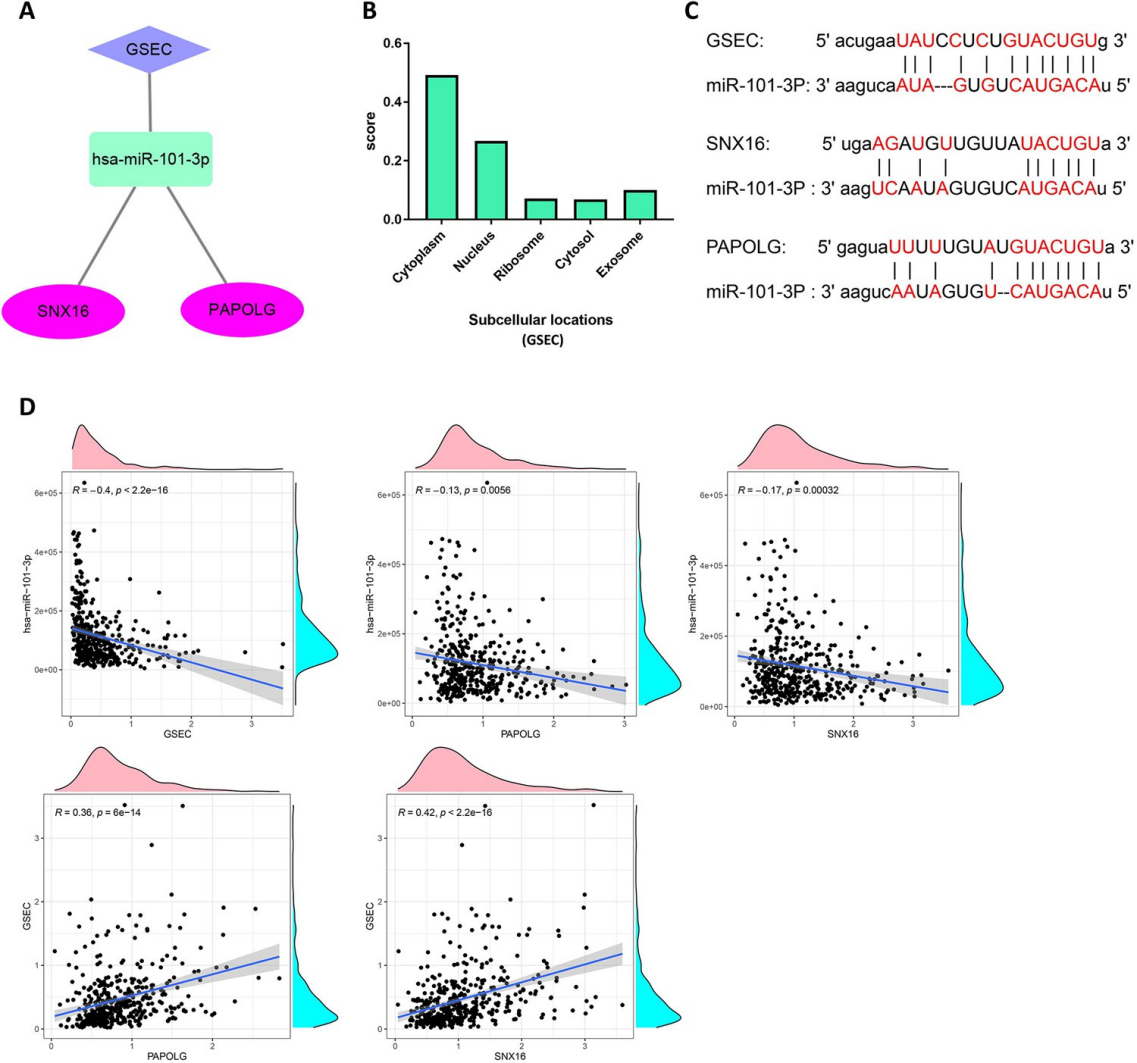
Construction of ceRNA network axis and correlation analysis. (A) ceRNA network axis GSEC/miR-101-3p/SNX16/PAPOLG. (B) Prediction of GSEC localization in cells using lncLocator. (C) ENCORI database prediction of the action sites of miR-101-3p with GSEC, the miR-101-3p with SNX16, PAPOLG, respectively. (D) Correlation analysis between these four genes (GSEC, miR-101-3p, SNX16, PAPOLG) based on TCGA data.

**Fig 8:**
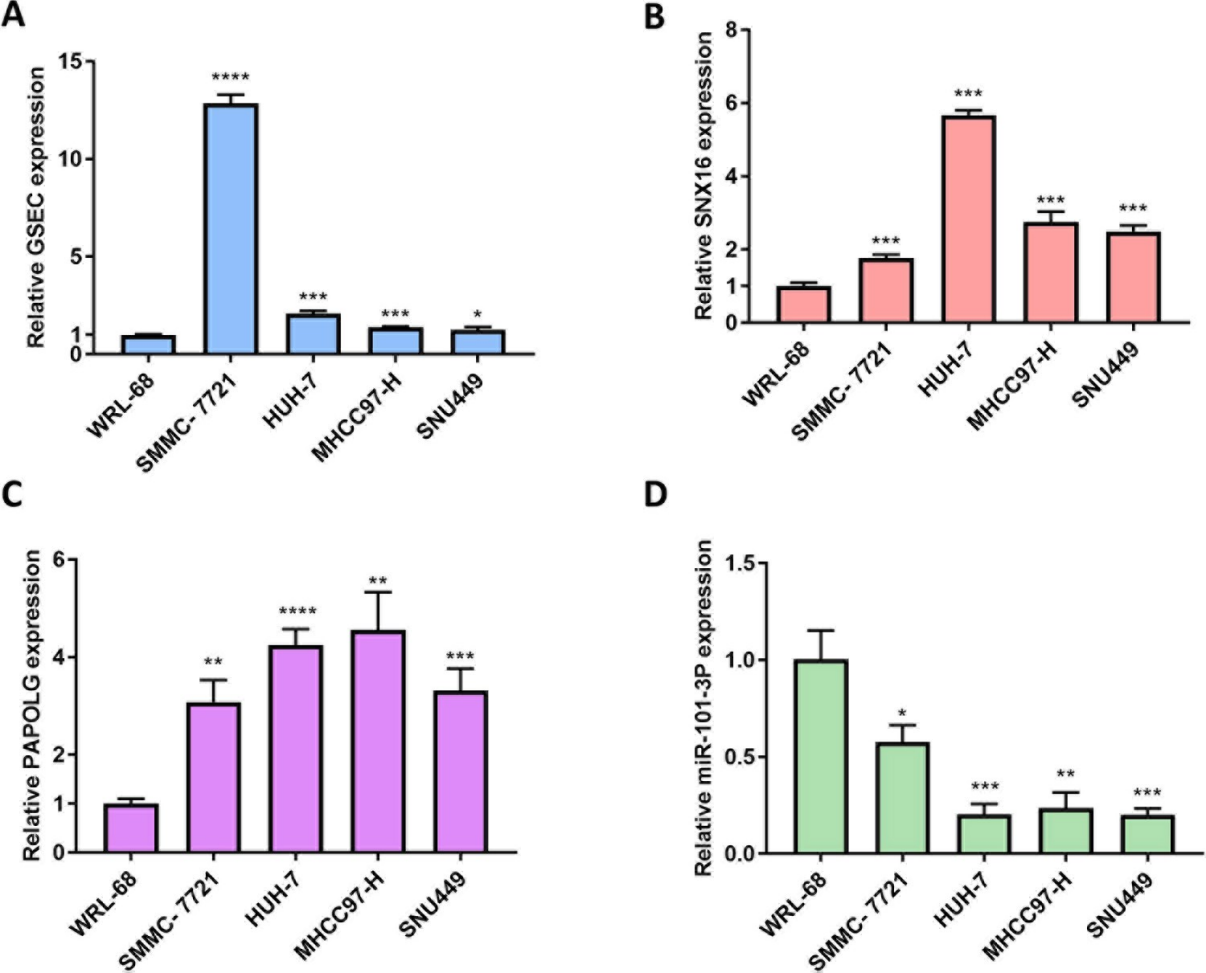
Relative expression of GSEC, SNX16, PAPOLG, miR-101-3p in SMMC-7721, HUH-7, MHCC97-H and SNU449 HCC cell lines and normal hepatocytes (WRL-68) cell lines. The mRNA levels of GSEC (A), SNX16 (B), PAPOLG (C), and miR-101-3p (D) in different cell lines. The experiments were performed independently for three times. P < 0.05 (*), P < 0.01 (**), P < 0.001 (***), P < 0.0001 (****).

Through the above analysis, we used mRNA-miRNA-lncRNA as a model to finally construct the ceRNA network axis GSEC/miR-101-3p/SNX16/PAPOLG (Fig 7A). In addition, the cellular localization of lncRNA affected the mechanism of action, and lncLocator analyzed the localization of GSEC in cells. As shown in Fig 7B, GSEC was mainly localized in the cytoplasm. Therefore, GSEC can act as an endogenous ceRNA to compete for binding miR-101-3p to adjust the expression of SNX16 and PAPOLG. The ENCORI database predicts the sites of action between GSEC and miR-101-3p and between miR-101-3p and SNX16 and PAPOLG (Fig 7C). Correlation analysis between GSEC/miR-101-3p/SNX16/PAPOLG axes indicated that miR-101-3p was negatively correlated with GSEC and SNX16/PAPOLG, while GSEC was positively correlated with SNX16 and PAPOLG, respectively (Fig 7D). In order to validate the bioinformatics results, the expression levels of GSEC, SNX16, PAPOLG, and miR-101-3p were measured using qRT-PCR in five cell lines, including four HCC cells (SMMC-7721, HUH-7, MHCC97-H and SNU449) and immortalized human hepatocyte WRL-68 cells. The results indicated that GSEC, SNX16 and PAPOLG were highly expressed in SMMC-7721, HUH-7, MHCC97-H and SNU449 HCC cell lines, while the low expression level of miR-101-3p was lower in four HCC cell lines (Fig 8). Therefore, the GSEC/miR-101-3p/SNX16/PAPOLG axis was used as a model for subsequent analysis.

### Construction of GSEC/miR-101-3p/SNX16/PAPOLG axis prognostic model

**Fig 9:**
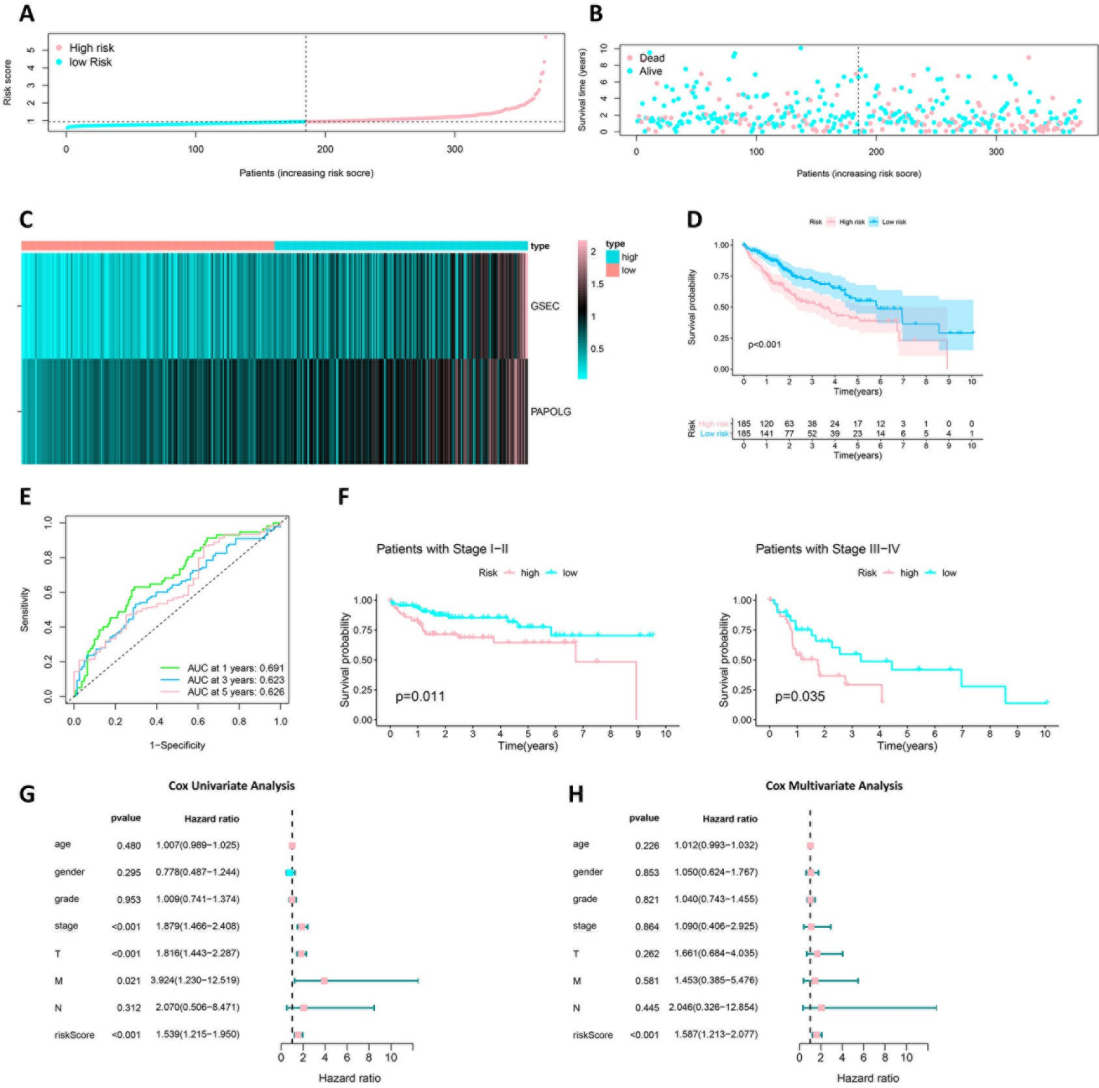
Prognostic value of risk model for GSEC and PAPOLG. (A) Risk curve for each sample based on risk value. (B) Scatter plot for each sample based on risk value. (C) Heat map showing expression of GSEC and PAPOLG in high and low risk. (D) Survival analysis of patients with high-risk HCC and low risk HCC based on risk model. (E) Risk model at 1 year (AUC = 0.691), 2-year (AUC = 0.623), and 3-year (AUC = 0.626) ROC curves. (F) Survival curves of HCC patients with TNM stages I-II (n = 162) and III-IV (n = 72). (G) Cox single-factor regression analysis of the risk model. (H) Cox multi-factor regression analysis of the risk model.

Multifactorial COX analysis was performed on GSEC, miR-101-3p, SNX16 and PAPOLG to construct the best risk prognostic model according to the Akaike information criterion (AIC = 1320.94). GSEC and PAPOLG were constructed as the best risk prognostic models (Risk value = 0.49t*GSEC-expression+0.40*PAPOLG-expression). The increased mortality in HCC patients depended on the increased risk score (Fig 9A and 9B), and GSEC and PAPOLG expression was upgraded in the high-risk group (Fig 9C). Survival analysis indicated that the overall survival of HCC patients in the low-risk group was significantly higher than that in the high-risk group (Fig 9D). In addition, the ROC curves 1-year AUC = 0.691, 3-year AUC = 0.623, and 5-year AUC = 0.626, indicating that the constructed risk model had high sensitivity and specificity (Fig 9E). The risk model was then divided into two groups of TNM stage Ⅰ-Ⅱ and stage Ⅲ-Ⅳ for survival analysis. As shown in Fig 9F, the survival of the high-risk group in both stage Ⅰ-Ⅱ and stage Ⅲ-Ⅳ was significantly smaller than that of the low-risk group (p<0.05). Therefore, we considered the TNM stage as a potential prognostic factor for the risk model. In the univariate COX regression analysis, TNM stage, depth of invasion, metastatic distance, and value at risk with was overall survival of HCC were prognostic factors in this risk model (p<0.05) (Fig 9G). In the multifactorial Cox regression analysis (Fig 9H), only the risk value was statistically significant (p<0.05). The results suggested that GSEC/miR-101-3p/SNX16/PAPOLG axis might be a significant prognostic significant role for HCC.

### GSEA and immune-infiltration analysis

**Fig 10:**
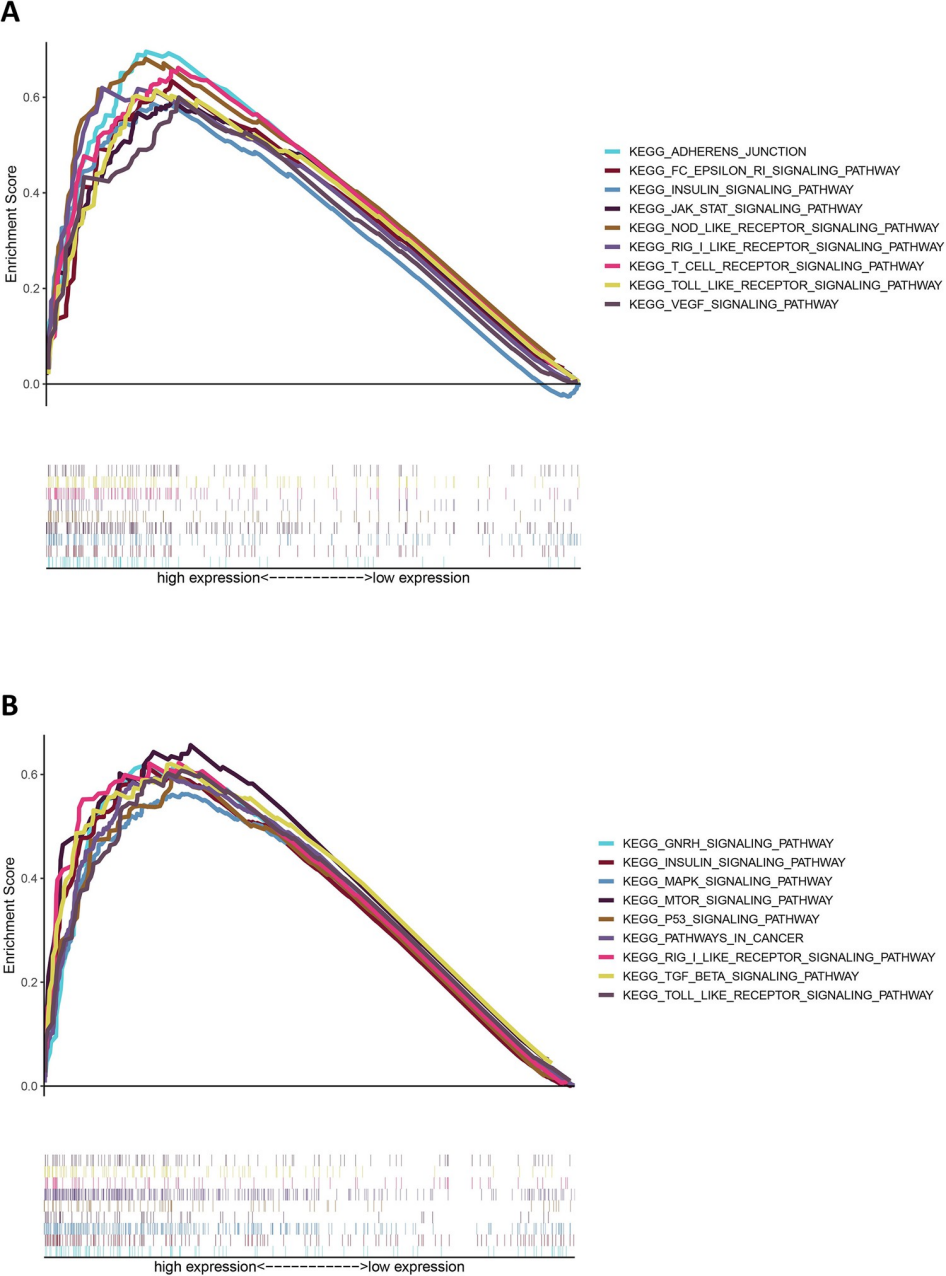
GSEA predicts SNX16 and PAPOLG related KEEG pathways. Pathways enriched in the SNX16 (A) and PAPOLG (B) high expression group, the enriched pathways are tumor and immune related. NOM < 0.01, FDR < 0.05.

**Fig 11:**
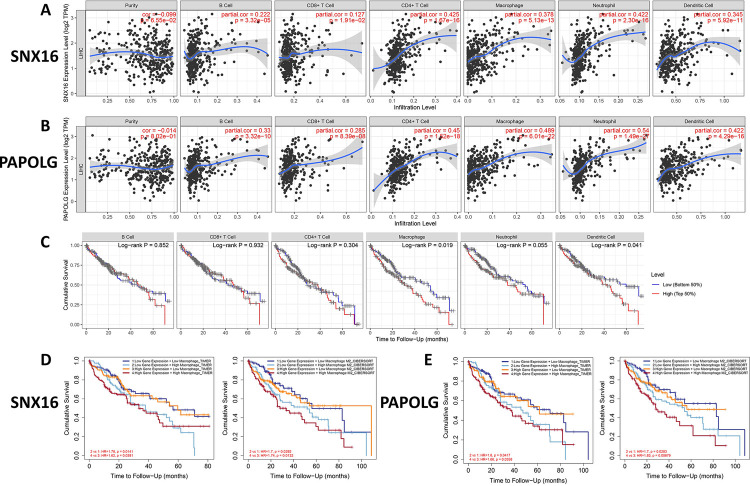
Correlation analysis of SNX16 and PAPOLG expression with immune infiltration. Correlation of SNX16 (A) and PAPOLG (B) expression with the level of immune cell infiltration. (C) Survival curves to analyze the relationship between six types of immune cell infiltration and overall survival of patients with HCC. Kaplan-Meier survival curves to analyze the effect of SNX16 (D) and PAPOLG (E) expression with macrophages and M2 macrophages on the overall survival of patients with HCC.

To predict the relevant pathways for SNX16 and PAPOLG enrichment, we performed GSEA on SNX16 and PAPOLG high expression groups. As shown in Fig 10A and 10B, SNX16 and PAPOLG high expression groups were mainly enriched in tumor and immune-related pathways (S4 and S5 Tables). We then used TIMER to estimate the relation between SNX16 and PAPOLG expression and immune cells and found that SNX16 and PAPOLG expression positively related with immune cells (Fig 11A and 11B). In addition, we assessed the prognostic impact of immune cell infiltration in HCC patients and found that macrophage infiltration was related to poor prognosis in HCC patients with survival time less than 75 months (Fig 11C). We speculated whether the expression of SNX16 and PAPOLG together with macrophages affects the prognosis of HCC patients, and we subsequently assessed whether the expression of SNX16 and PAPOLG together with macrophages and its subtypes affects the prognosis of HCC patients. As shown in Fig 11D, the overall survival of group 2 (low SNX16 expression + High Macrophage) was significantly lower than that of group 1 (low SNX16 expression + Low Macrophage), and that of group 3 (High SNX16 expression +Low Macrophage) was significantly lower than that of group 4 (High SNX16 expression + High Macrophage). The expression of M2 macrophages with SNX16 had the same results on the survival of HCC. The expression of PAPOLG with macrophages and their isoforms (M2) also had similar results on the prognosis of patients with HCC (Fig 11E). Thus, immune infiltrating cells influenced the prognosis of the GSEC/miR-101-3p/SNX16/PAPOLG axis on HCC patients.

### Correlation of SNX16 and PAPOLG expression with macrophage subtype markers

**Fig 12:**
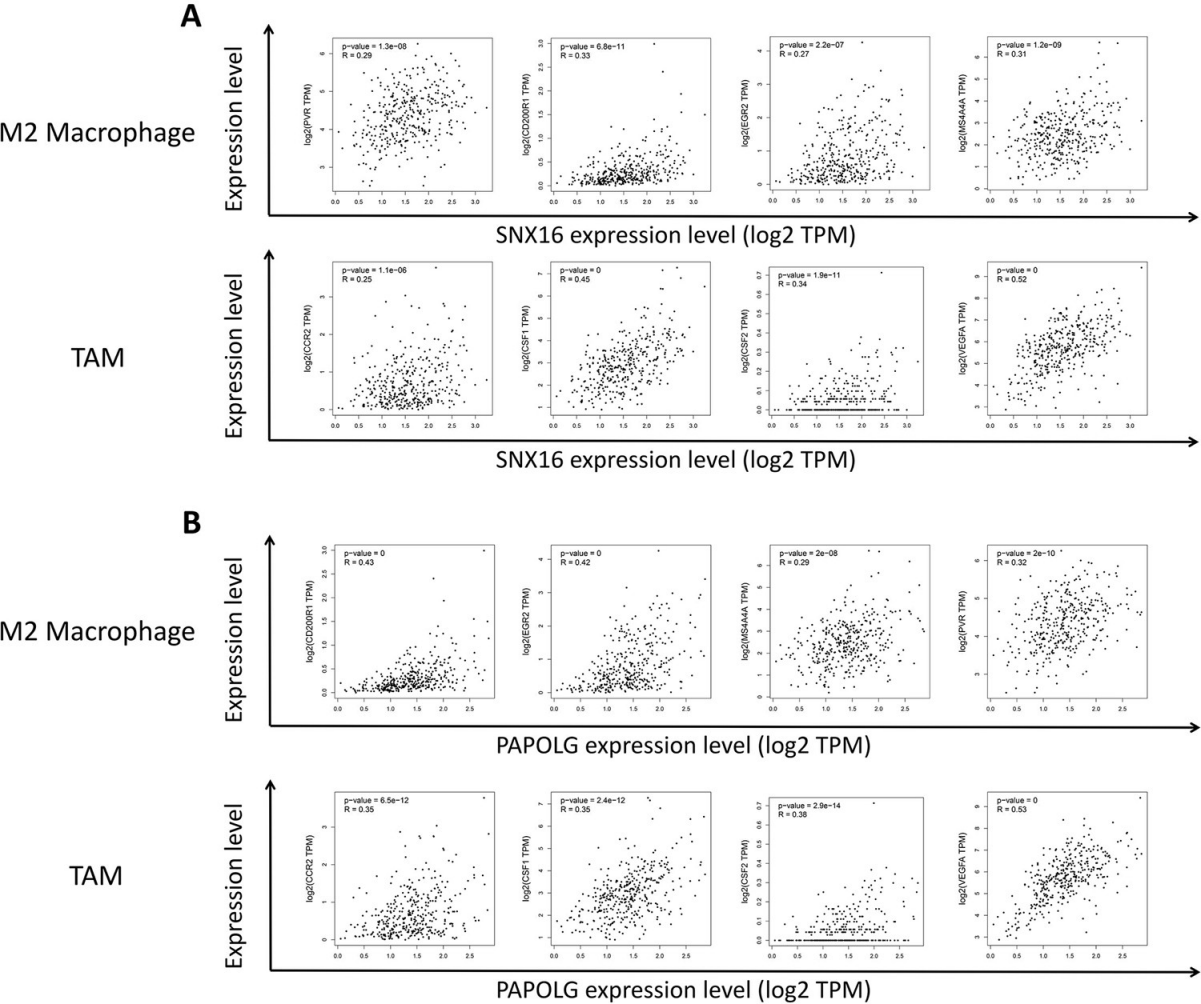
Effect of SNX16 and PAPOLG expression on macrophage polarization in HCC. Scatter plot of SNX16 (A) and PAPOLG (B) expression correlation with M2 macrophages (PVR, CD200R1, EGR2, MS4A4A) and tumor-associated macrophage (TAM) (CCR2, CSF1, CSF2, VEGFA) gene markers.

The expression of SNX16 and PAPOLG positively related with macrophages and the expression of SNX16 and PAPOLG together with macrophages and other subtypes (M2) influenced the prognosis of HCC patients. We hypothesized that the expression of SNX16 and PAPOLG might affect the polarization of macrophage M2. The GEPIA database was used to analyze the correlation of SNX16 and PAPOLG expression with M2 macrophage markers and their tumor-associated macrophages (TAMs) markers, as shown in Fig 12A and 12B. M2 macrophage markers (such as PVR, CD200R1, EGR2 and MS4A4A) were positively related with the expression of SNX16 and PAPOLG. TAMs markers (such as CCR2, CSF1, CSF2 and VEGFA) were positively related with the expression of SNX16 and PAPOLG. The GSEC/miR-101-3p/SNX16/PAPOLG axis might affect M2 macrophage polarization and differentiation of macrophages to TAMs, which might play an important role during the development of HCC.

## Discussion

In the present study, the result suggested that SNX16 was strongly correlated with PAPOLG. And it was the first time to identify the highly expressed levels of SNX16, PAPOLG and GSEC, and the lowly expression level of miR-101-3p in HCC, which were all related with the TNM stage of HCC patients. In addition, high expression of SNX16, PAPOLG and GSEC was related with poor prognosis in HCC patients, and high expression of miR-101-3p was associated with good prognosis in HCC patients. In the constructed risk model, the overall survival of HCC patients in the low-risk group was strongly higher than the overall survival of the high-risk group, and in addition, the survival of the high-risk group in both stage I-II and stage III-IV was strongly smaller than that of the low-risk group. Through cell experiment verification, the levels of GSEC, SNX16, and PAPOLG mRNAs were highly expressed in SMMC-7721, HUH-7, MHCC97-H, and SNU449 HCC cell lines, while the expression level of miR-101-3p was significantly lower in those HCC cell lines, which is consistent in HCC patient samples through bioinformatics analysis. The final construction of the ceRNA network axis GSEC/miR-101-3p/SNX16/PAPOLG related with HCC prognosis.

Over the past few decades, significant progress has been made in early diagnosis, surgical treatment, and liver transplantation, so the morbidity and mortality of HCC have been significantly reduced. However, the prognosis of HCC remains poor, and the mortality rate is still high, and the mechanism of HCC development is still unclear [27]. It is crucial to develop efficient treatment methods and to elucidate the molecular mechanisms of HCC. According to the ceRNA network hypothesis, lncRNA could act as an endogenous molecular sponge competing for binding miRNA and regulating the expression level of mRNA [9]. The previous report showed that HOXD-AS1 could competitively bind miR-130a-3p and inhibit the degradation of the target gene SOX4, thus promoting the metastasis of HCC cells [28]. And the result suggested that lncRNA BCAR4 could up-regulate the expression of miR-1261 by inhibiting the expression of ANAPC11 (Anaphase Promoting Complex Subunit 11), thus promoting the development of HCC [29]. However, ceRNA network of HCC are still unclear. To the best of our knowledge, the mRNA-miRNA-lncRNA model was the first time to be uses to analyze specific prognosis-related ceRNA networks in HCC. In this study, a new mRNA-miRNA-lncRNA (GSEC/miR-101-3p/SNX16/PAPOLG) network axis was established, among which each RNA plays an important prognostic role in HCC.

In this study, the microRNA of miR-101-3p and the lncRNA of GSEC were identified associated to SNX16 and PAPOLG. Previously in the study of Li *et al*., the results on bioinformatics and meta-analysis indicated that miR-101-3p expression was downregulated in HCC, and overexpression of miR-101-3p was able to inhibit the proliferation and promote apoptosis of HCC cells [30].Currently, miR-101-3p has been widely studied in colorectal cancer, acting as an oncogene in colorectal cancer. The published reported showed that the knockdown of miR-101 could inhibit the proliferation and invasive ability of colorectal cancer cells via activating the Wnt/β-catenin signaling pathway [31]. Another study reported that the overexpression of miR-101-3p could reverse the pro-tumorigenic effect of cyclic RNA VAPA, which promoted colorectal cancer development [32]. GSEC (G-quadruplex forming sequence containing lncRNA) is a novel lncRNA localized in the cytoplasm [33]. The knockdown of GSEC was reported to reduce motility activity in colon cancer cells, and in addition, GSEC could bind DHX36 to reduce its activity to promote colon cancer development [33]. On the other hand, the overexpression of GSEC could promote osteosarcoma progression by inhibiting the miR-588/EIF5A2 signaling pathway [34].

In this study, the GSEC/miR-101-3p/SNX16/PAPOLG axis was constructed as a ceRNA network axis, which could be a new important prognostic factor associated with HCC prognosis. Moreover, the result of gene set enrichment analysis suggested that SNX16 and PAPOLG were mainly enriched in tumor- and immune-related pathways. As we known, the immune infiltration could affect the prognosis of HCC patients [35–37]. The current result of immune infiltration associated with SNX16 and PAPOLG in this study showed that SNX16 and PAPOLG expression positively correlated with six immune cells, and that macrophage infiltration was related with poor prognosis in HCC patients. We subsequently demonstrated that SNX16 and PAPOLG expression together with macrophages and their macrophage subtypes (M2) were co-influential to HCC prognosis of patients. We further explored the correlation of SNX16 and PAPOLG expression with M2 macrophage markers and their TAMs markers. The results showed that SNX16 and PAPOLG expression were positively correlated with M2 macrophage markers and their TAMs markers. M2 macrophages have a role in promoting tumor development, and M2 macrophages promote angiogenesis, promote tumor cell metastasis, and help tumor cells evade immune recognition [38, 39]. Several studies have shown that M2 macrophages have significant effects on the development of HCC. For example, lncRNA LINC00662 could activate Wnt/β-linked protein signaling in macrophages and further promoted M2 macrophage polarization, thereby promoting the value-added and metastasis of HCC cells and inhibiting apoptosis [40]. The co-culture of M2 macrophages with HCC cells could increase the metastatic capacity and EMT marker expression of HCC cells and lead to increased expression of the oncogene TLR4, further promoting the migration of HCC cells [41]. In addition, a number of studies have published that TAMs promote migration [42], proliferation [43], and angiogenesis [44] of cancer cells and are also related with the prognosis of HCC patients [45]. Therefore, we hypothesized that the GSEC/miR-101-3p/SNX16/PAPOLG axis might influence M2 macrophage polarization and differentiation of macrophages to TAMs and thus have an impact on the development of HCC.

## Conclusions

In summary, the GSEC/miR-101-3p/SNX16/PAPOLG axis was constructed to analyze to HCC prognosis and immune infiltration by the "mRNA-miRNA-lncRNA" model, which could help us better understand the development mechanism of HCC. It might be a new theoretical basis for the study of molecular mechanisms and immunotherapy of HCC.

## Supporting information

S1 Table Clinicopathological characteristics statistics of HCC patients (mRNA/LncRNA) from TCGA. (DOCX)

S2 Table Clinicopathological characteristics statistics of HCC patients (miRNA) from TCGA. (DOCX)

S3 Table SNX16 and PAPOLG immunohistochemical samples. (DOCX)

S4 Table Tumor and immune related pathways of SNX16. (DOCX)

S5 Table Tumor and immune related pathways of PAPOLG. (DOCX)

S1 Fig SNX16 expression on protein level was verified in Human Protein Atlas (HPA) database. (TIF)

S2 Fig PAPOLG expression on protein level was verified in Human Protein Atlas (HPA) database. (TIF)

S3 Fig Identification of miRNAs with low expression and good prognosis in HCC. (TIF)

S4 Fig Identification of lncRNAs with high expression and poor prognosis in HCC. (TIF)
