# Supplementary material for: Comprehensive analysis of GSEC/miR-101-3p/SNX16/PAPOLG axis in hepatocellular carcinoma
Source: PLoS One. 2022 Apr 28;17(4):e0267117. doi: 10.1371/journal.pone.0267117 (PMC9049542; doi:10.1371/journal.pone.0267117)
Supplement: S1 Table — (DOCX) [file pone.0267117.s001.docx]

**S1 Table. Clinicopathological characteristics statistics of HCC patients (mRNA/LncRNA) from TCGA.**

| Clinical characteristics | number | |
| --- | --- | --- |
| Age at diagnosis (y)  Gender  Stage  Grade  Invasion depth(T)  Distant metastasis(M)  Lymph node metastasis(N) | young age (<=60)  old age (>60)  Male  Female  Ⅰ  Ⅱ  Ⅲ  Ⅳ  G1  G2  G3  G4  T1  T2  T3  T4  M0  M1  N0  N1 | 131  104  74  161  113  50  67  5  31  103  93  8  115  52  58  10  231  4  231  4 |
