# Supplementary material for: Comprehensive analysis of GSEC/miR-101-3p/SNX16/PAPOLG axis in hepatocellular carcinoma
Source: PLoS One. 2022 Apr 28;17(4):e0267117. doi: 10.1371/journal.pone.0267117 (PMC9049542; doi:10.1371/journal.pone.0267117)
Supplement: S2 Table — (DOCX) [file pone.0267117.s002.docx]

**S2 Table. Clinicopathological characteristics statistics of HCC patients (miRNA) from TCGA.**

| Clinical characteristics | number | |
| --- | --- | --- |
| Age at diagnosis (y)  Gender  Stage  Grade  Invasion depth(T)  Distant metastasis(M)  Lymph node metastasis(N) | young age (<=60)  old age (>60)  Male  Female  Ⅰ  Ⅱ  Ⅲ  Ⅳ  G1  G2  G3  G4  T1  T2  T3  T4  M0  M1  N0  N1 | 133  104  73  164  115  50  67  5  31  102  95  9  117  52  58  10  233  4  233  4 |
