# Supplementary material for: Comprehensive analysis of GSEC/miR-101-3p/SNX16/PAPOLG axis in hepatocellular carcinoma
Source: PLoS One. 2022 Apr 28;17(4):e0267117. doi: 10.1371/journal.pone.0267117 (PMC9049542; doi:10.1371/journal.pone.0267117)
Supplement: S3 Table — (DOCX) [file pone.0267117.s003.docx]

**S3 Table. SNX16 and PAPOLG immunohistochemical samples**

| # in image | HPA number | Tissue type | ID | Age | Sex | Staining |
| --- | --- | --- | --- | --- | --- | --- |
| 1 | HPA024817 | normal | 3402 | 54 | Female | Not detected |
| 2 | HPA024817 | normal | 3222 | 63 | Female | Not detected |
| 3 | HPA024817 | tumor | 2177 | 58 | Female | High |
| 4 | HPA024817 | tumor | 3196 | 67 | Male | High |
| 5 | CAB017795 | normal | 2429 | 55 | Male | Not detected |
| 6 | CAB017795 | normal | 3378 | 73 | Male | Not detected |
| 7 | CAB017795 | tumor | 2766 | 73 | Female | Low |
| 8 | CAB017795 | tumor | 3196 | 65 | Male | Low |
