# Supplementary material for: Comprehensive analysis of GSEC/miR-101-3p/SNX16/PAPOLG axis in hepatocellular carcinoma
Source: PLoS One. 2022 Apr 28;17(4):e0267117. doi: 10.1371/journal.pone.0267117 (PMC9049542; doi:10.1371/journal.pone.0267117)
Supplement: S4 Table — (DOCX) [file pone.0267117.s004.docx]

**S4 Table. Tumor and immune related pathways of SNX16**

| KEGG Pathy | NES | NOM p-val | FDR q-val |
| --- | --- | --- | --- |
| KEGG_ADHERENS_JUNCTION | 2.05 | 0 | 0.001 |
| KEGG_INSULIN_SIGNALING_PATHWAY | 2.04 | 0 | 0.001 |
| KEGG_RIG_I_LIKE_RECEPTOR_SIGNALING_PATHWAY | 2.01 | 0 | 0.002 |
| KEGG_NOD_LIKE_RECEPTOR_SIGNALING_PATHWAY | 2.01 | 0 | 0.002 |
| KEGG_TOLL_LIKE_RECEPTOR_SIGNALING_PATHWAY | 1.97 | 0 | 0.003 |
| KEGG_JAK_STAT_SIGNALING_PATHWAY | 1.97 | 0 | 0.002 |
| KEGG_VEGF_SIGNALING_PATHWAY | 1.96 | 0 | 0.002 |
| KEGG_T_CELL_RECEPTOR_SIGNALING_PATHWAY | 1.94 | 0 | 0.003 |
| KEGG_FC_EPSILON_RI_SIGNALING_PATHWAY | 2.06 | 0 | 0.001 |
